# The Structure and Clinical Roles of MicroRNA in Colorectal Cancer

**DOI:** 10.1155/2016/1360348

**Published:** 2016-12-28

**Authors:** Linwensi Zhu, Jingyuan Fang

**Affiliations:** Division of Gastroenterology and Hepatology, Key Laboratory of Gastroenterology and Hepatology, Ministry of Health and State Key Laboratory for Oncogenes and Related Genes, Renji Hospital, Shanghai Jiao Tong University, Shanghai Institution of Digestive Disease, 145 Middle Shandong Road, Shanghai 200001, China

## Abstract

Colorectal cancer (CRC) is one of the most prevalent types of malignancies, particularly among individuals aged between 50 and 75. The global incidence of CRC has been steadily on the rise due in no small part to an aging population and a shift in lifestyle as well as eating habits. MicroRNAs are a group of small, noncoding, and endogenous RNA molecules that have recently emerged as key players in a broad range of pathological pathways. Moreover, dysregulation of microRNAs has been implicated in cancer development and metastasis. This review is intended to provide a brief overview of the structure, functions, and clinical roles of microRNAs. In particular, the review will focus on the discovery, the underlying mechanistic roles, and the diagnostic as well as therapeutic potentials of CRC-specific miRNAs.

## 1. Introduction

Colorectal cancer (CRC) is the third most common type of cancer and ranks fourth in the number of cancer-related deaths around the world [1]. Like any other cancers, the pathogenesis of CRC is complex and characterized by multiple gene mutations and dysregulated signaling networks, with significant genetic variations among individual patients. Recently, mounting evidence suggests that microRNAs (miRNAs), which are small, noncoding single-stranded RNAs ranging from 19 to 22 nucleotides in length and highly conserved throughout evolution, play important roles in modulating gene expression and various biological processes through targeting mRNAs. The first miRNA, miRNA-lin-4, was identified in 1993 in* Caenorhabditis elegans* as a crucial regulatory factor that governs its larval development [2]. Since then, over thousands of miRNAs have been identified in plants, animals, and viruses [3]. In mammals, the 5′-terminal region of the mature miRNA features a short and highly conserved segment of sequence, often referred to as the “seed region,” which can complement with the 3′ untranslated region (UTR) of the target mRNA [4]. The interaction then leads to the recognition and ultimately the functional suppression of the target mRNA by the RNA-induced silencing complex (RISC) through translational repression, direct degradation, or irreversible decay induced by deadenylation-dependent decapping [5, 6]. Due to these biological events, studies have now increasingly considered miRNAs as key players in numerous pathological, including oncogenic, processes [2, 7]. These findings have provided a strong incentive for the identification of CRC-associated miRNAs that can be used to develop novel diagnostic and treatment solutions.

## 2. Overview of miRNA Expression in CRC

The initial evidence implicating miRNAs in CRC pathogenesis was provided by Michael et al.'s study, which indicated that miRNA-143 and miRNA-145 were consistently downregulated in cancerous colorectal neoplasia [8]. Since then, increased understanding of mRNA biogenesis and technological advances in areas such as bioinformatics and microarray analysis have allowed novel CRC-related miRNAs to be identified at an accelerated pace.

### 2.1. Tissue miRNA Expression in CRC Patients

Tumor tissue is the most important source for the identification of CRC-related miRNAs. In addition to above-mentioned miRNA-143 and miRNA-145, miRNA-21 expression in CRC tissues was noted in multiple studies to progressively increase with tumor stage [9, 10]. Liu et al. and Oue et al. independently reported the existence of a significant correlation between elevated tissue miRNA-21 level and poor chemotherapeutic outcome in CRC patients [11–28]. In addition, miRNA-21 was upregulated in stromal cells of CRC tissues and associated with shorter remission after surgical treatment [13]. Elevated tissue expression of miRNA-31 has also been observed [9–11, 14], particularly in patients with advanced CRC [11, 14]. A clinical examination of 52 paired CRC tissue samples by Xu et al. suggested a positive correlation between the expression level of miRNA-135b and the degree of tumor cell differentiation grade [10]. These results were echoed by two other miRNA profiling studies published in the same year [15, 16]. Akçakaya et al. compared the miRNA expression profiles between CRC patients with long- and short-term survival. Their results suggest that increased expression of miRNA-185 and diminished level of miRNA-133b in cancerous tissues could serve as prognostic indicators [17].

Evidence emerges that miRNAs often exist in polycistronic clusters and regulate oncogenic pathways in a synergistic fashion. A prime example is the highly conserved and well-characterized miRNA-17~92 cluster, which encodes six miRNAs including miRNA-17, miRNA-18a, miRNA-19a, miRNA-20a, miRNA-19b, and miRNA-92a [18]. overexpression of miRNA-17~92 and its individual members has been consistently observed in CRC [19, 20]. Increased level of miRNA-17 in colon cancer tissues was reported to be a strong prognostic indicator for poor clinical outcome [21]. Similarly, upregulation of miRNA-92a expression was confirmed in CRC tissues and correlated negatively with overall survival [22]. overexpression of miRNA-20a, together with miRNA-21, miRNA-106a, miRNA-181b, and miRNA-203, was confirmed in Schetter et al.'s microarray profiling of colon adenocarcinoma tissues obtained from a cohort of 84 patients [23].

### 2.2. Serum miRNA Expression in CRC Patients

Accurate quantitation of circulatory miRNA levels in blood serum allows early detection and provides a powerful tool for monitoring tumor progression in CRC patients. Comparing the expression levels of multiple miRNAs between 88 patients with primary CRC and 11 healthy adults [24], Ogata-Kawata and colleagues revealed that let7a, miRNA-1229, miRNA-1246, miRNA-150, miRNA-21, miRNA-223, and miRNA-23a were significantly upregulated in patients. However, the expression of these miRNAs was attenuated following surgical tumor resection [24]. In another study, the blood plasma levels of miRNA-24, miRNA-320a, and miRNA-423-5p were shown by qRT-PCR to be much lower in a cohort of 223 CRC patients than in healthy individuals [25]. Again, surgical removal of the tumor restored the expression of all three miRNAs back to normal [25]. Hu et al. discovered that the serum levels of miRNA-1914^*∗*^ and miRNA-1915 differed sharply between CRC patients who showed good clinical response to treatment and those that responded poorly [26]. Further investigation revealed that both miRNA-1914^*∗*^ and miRNA-1915 could target the 3′-UTR of nuclear factor I/X (NFIX). Subsequent in vitro cellular assays demonstrated that overexpression of miRNA-1914^*∗*^ and miRNA-1915 could act to resensitize chemoresistant tumor cells to common therapeutic drug molecules such as fluorouracil and oxaliplatin, suggesting that they have potential to boost the clinical efficacy of chemotherapy for CRC treatment.

### 2.3. Fecal miRNA Expression in CRC Patients

Identification and analysis of fecal miRNAs have gained attraction in recent years as stool analysis is increasingly being used as a noninvasive diagnostic tool for early CRC screening. Cells that are shed from CRC tissues can provide valuable genetic and epigenetic information to facilitate tumor detection [27]. Compared to other common nucleotide-based molecules, miRNAs are considerably more stable and RNase-resistant, making them particularly well-suited targets in stool analysis. The feasibility of isolating fecal RNAs and quantifying mature miRNAs with RT-PCR was initially demonstrated in Ahmed et al.'s study, in which 7 miRNAs, including miRNA-320, miRNA-126, miRNA-484-5p, miRNA-143, miRNA-145, miRNA-16, and miRNA-125b, were downregulated in stool samples collected from CRC patients when compared to those of healthy individuals [28]. In another study conducted by Link and colleagues, unique CRC-associated miRNA signatures, which could serve as novel diagnostic biomarkers, were identified by comparing the fecal miRNA expression patterns in CRC patients and normal volunteers by RT-PCR and microarray analysis [29]. A comparison of fecal miRNA profiles between 198 patients with CRC, 199 with polyps, and 198 controls using a miRNA expression array found the expression of miRNA-221 and miRNA-18a to steadily increase with tumor initiation and progression [30]. Using an analogous approach, Wu et al. observed a substantial upregulation of miRNA-135b in stool samples from CRC patients, but not from those with inflammatory bowel disease (IBD), which shares many similar symptoms with CRC and is a common cause for misdiagnosis [31]. In addition, decreased fecal levels of miRNA-29, miRNA-223, and miRNA-224 have also been detected in CRC patients. It is noteworthy that fecal miRNA-29 was found to be significantly more abundant in rectum cancer patients than those with colon cancer, suggesting the feasibility of using differential miRNA expression patterns as cancer fingerprints [32].

## 3. Implication of miRNAs in the Development and Progression of CRC

The fact that the complementation between miRNA and its target mRNA only spans a very short segment of nucleotides leads to a combinatorial regulatory paradigm, where one miRNA can target multiple mRNAs and one mRNA can be controlled by many different miRNAs [33]. This highlights the extreme complexity of various miRNA-dependent regulatory networks. In fact, oncogenic and tumor-suppressive miRNAs frequently undergo opposing regulatory patterns in CRC patients.

The mechanistic roles of miRNA-145 and miRNA-143, the first two miRNAs identified to be associated with CRC pathogenesis, have been widely examined in a host of studies. Gregersen et al. conducted microarray analysis to probe the gene expression profiles in the colon cancer cell line DLD-1 overexpressing miRNA-145 and identified multiple downstream mRNA targets. Among them, YES and signal transducer and activator of transcription 1 (STAT1), both of which have previously been associated with cancer development [34], were experimentally verified by qRT-PCR [35]. In Sachdeva and Mo's study, knockdown of miRNA-145 was found to derepress the metastasis gene mucin 1 (MUC1), a well-established prognostic biomarker for CRC and many other types of cancer [36]. On the other hand, using microarray analysis followed by experimental validation, the expression levels of miRNA-143 and V-Ki-ras2 Kirsten rat sarcoma viral oncogene homolog (KRAS) were shown to be negatively correlated with each other [37]. This finding was immediately followed by the identification of DNA methyltransferases 3A as an additional target of miRNA-143 [38]. Very recently, Zhang and colleagues suggested that the ability of miRNA-143 to impede CRC cell growth and invasion might involve its targeting of metastasis-associated in colon cancer-1 (MACC1) [39].

The downstream targets of other CRC-associated miRNAs have also been uncovered. A recent study showed that transfection with miRNA-382 mimics significantly impaired the proliferation and migration of CRC cells. The binding of the 3′-UTR of nuclear receptor subfamily 2 (group F, member 2) (NR2F2) to miRNA-382 was subsequently confirmed by luciferase report assay [40]. As NR2F2 expression was shown to be markedly upregulated in CRC tissues, it is likely that the miRNA-382/NR2F2 regulatory pair could serve as a therapeutic tool for targeted CRC treatment. The expression of miRNA-152 was found to be downregulated in CRC cell lines and inversely correlated to their metastatic propensity [41]. Restoring miRNA-152 expression to normal levels dramatically suppressed tumor cell proliferation and migration while simultaneously resulting in increased apoptosis. Subsequent investigation revealed that miRNA-152 is able to inhibit phosphoinositide-3-kinase regulatory subunit 3 (PIK3R3). In fact, it was discovered that direct activation of PIK3R3 led to CRC-suppressing effects similar to those observed due to miRNA-152 activation. Yang and colleagues confirmed that overexpression of miRNA-182 enhanced the proliferative and metastatic capabilities of CRC cells by targeting the 3′-UTR of special AT-rich sequence binding protein 2 (SATB2) and caused tumor cells to undergo epithelial-mesenchymal transition (EMT) [42].

The discovery of the miRNA-17~92 cluster has attracted intense interest in deciphering its downstream targets. Knockdown of miRNA-17~92 cluster increased the expression of antiangiogenic thrombospondin-1 (Tsp1) and connective tissue growth factor (CTGF), leading to reduced neovascularization in tumors originating from p53-null colonocytes [43]. Knockdown experiments performed on individual cluster members revealed that miRNA-19 and miRNA-18 were responsible for the suppression of Tsp1 and CTGF, respectively. Tsuchida and colleagues demonstrated that miRNA-92 could effectively protect colon cancer cells from apoptosis by targeting BCL-2-interacting mediator of cell death (BIM) [44]. Furthermore, miRNA-92 was shown to regulate the expression of phosphatase and tensin homolog (PTEN) in CRC tissues [45], which activated EMT and contributed to increased tumor proliferation and invasion. Taken together, these findings are consistent with the general picture of a vast and extremely intricate regulatory network that connects miRNAs with cancer development, progression, and metastasis.

## 4. Application of miRNAs in CRC Diagnosis and Treatment

### 4.1. Use of miRNAs for Early Detection of CRC

Currently, CRC screening of suspected individuals generally begins with a fecal occult blood test (FOBT) and/or a fecal immunochemical test (FIT) to capture microscopic blood in the digestive tract. While both tests are considered noninvasive and inexpensive, they often produce inaccurate results [46], which necessitate the use of colonoscopy to provide conclusive diagnosis. However, the performance of colonoscopy requires dietary changes and colon cleansing prior to the examination. Furthermore, there is a small but nonnegligible risk of infection, even injury [47]. To address these drawbacks, miRNAs have been proposed as potential candidates for the development of novel, effective CRC screening approaches that could simultaneously achieve high diagnostic accuracy and operational convenience. There are several underlying considerations that lend support to this proposal. First, miRNAs have been found in many studies to be stably present in serum and stool samples. This was demonstrated by Chen et al.'s finding that serum miRNAs in CRC patients displayed remarkable resistance to not only RNase digestion but also harsh procedures such as boiling, freeze-thaw cycles, and acid/alkaline treatment [48]. Similar observations were noted in Ahmed et al.'s aforementioned study on fecal miRNAs, even though the composition of stool is substantially more unfavorable for miRNA detection than that of blood [28]. Second, miRNA signatures are often highly unique in different tumors. In one pioneering study, Lu and colleagues created a training set consisting of 68 differentiated tumor specimens that could be correctly classified and developed a model system based on a differential analysis of their miRNA profiles. The model was shown to lead to correct grouping to tumor tissues according to their origins, including those from CRC. In addition, when used to predict the origin of 17 poorly differentiated malignancies, the miRNA-based tumor classification approach was found to be considerably more accurate than the conventional epigenetic method relying on mRNA biomarkers, highlighting the potential of using a relatively small subset of miRNAs (around 200) for cancer identification [49].

Based on these advantages, the diagnostic value of miRNAs for CRC detection was demonstrated in a number of studies. A meta-analysis of three microarray studies on differential miRNA expression patterns between CRC patients and healthy individuals identified 25 potential candidates, of which 15 were dysregulated in early-stage CRC tissues [50]. In particular, miRNA-195 and miRNA-20a were identified in all three studies. Ng et al. reported that the expression of miRNA-17-3p and miRNA-92a was significantly upregulated in patients diagnosed with CRC and lowered when their tumors were surgically removed [51]. miRNA-92a was then used in the diagnostic analysis of 180 independent plasma samples and produced satisfactory sensitivity (89%) and specificity (70%) values. Consistent with these results, miRNA-92a and miRNA-29a were identified by receiver operating (ROC) characteristic curve analysis to be reliable predictors of early-stage CRC when applied to a large validation group comprising 100 patients and 59 normal controls [52]. Both miRNA candidates were also shown in the same study to be able to serve as biomarkers for advanced adenoma.

### 4.2. miRNAs as Novel Targets in Therapies against CRC

The current lineup of cancer treatment methods, including surgery, chemotherapy, and radiotherapy, is far from adequate. Surgical removal of tumor requires a margin of resection and is generally impossible on cancerous tissues that are poorly differentiated or have metastasized. Meanwhile, chemotherapy and radiotherapy frequently incur severe side-effects on patients and are often thwarted by the emergence of drug- and radiation-resistant cancer cells. Consequently, miRNAs have been proposed as potential therapeutic targets for tumor treatment. One of the most significant advantages of miRNA-based therapeutics lies in the ability to simultaneously target multiple effectors and pathways, thereby maximizing tumor specificity while minimizing the likelihood of resistance. In addition, miRNAs involved in the development of tumor resistance to chemotherapeutic agents or radiation can be directly targeted.

One of the strategies employed in miRNA-based therapeutics involves the inhibition of carcinogenic miRNAs. The potential clinical value of miRNA-21 for treating colon cancer was discussed in several literature reports [53, 54]. Suppression of miRNA-21 was revealed to resensitize chemotherapy-resistant colon cancer cells to drugs such as fluorouracil, oxaliplatin, and difluorinated curcumin [53]. In another study, knockdown of miRNA-451 was found to impede the migration and proliferation of CRC cell line HT-29 [55]. The observed antitumor effect was associated with the derepression of 5′ AMP-activated protein kinase (AMPK), which in turn led to the inhibition of both mammalian target of rapamycin complex 1 (mTORC1) and its regulatory target fascin actin-bundling protein 1 (FSCN1). Yannan and Haihang conducted microarray analysis on two CRC cell lines with drastically different metastatic capabilities and found miRNA-499-5p to play a pivotal role in tumor metastasis by targeting forkhead box protein O4 (FOXO4) and programmed cell death 4 (PDCD4) [56]. Disruption of miRNA-451 expression was confirmed to attenuate the invasion and migration of CRC cells, suggesting that it might serve as an oncogenic target in anticancer therapies.

As an alternative approach, several research groups sought to contain tumor proliferation and metastasis via boosting the expression of cancer-suppressing miRNAs. Wang et al. reported that miRNA-375 could directly target the 3′-UTR of the gene encoding phosphatidylinositol-4,5-bisphosphate 3-kinase, catalytic subunit alpha (PIK3CA), leading to the inhibition of the PI3K/Akt pathway [57]. Subsequent animal studies demonstrated that overexpression of miRNA-375 exerted therapeutic effect by impeding tumor growth [57]. Shi et al. noticed that miRNA-142-5p expression was significantly inhibited in stage III CRC tissues but was upregulated following transcatheter arterial infusion chemotherapy [58]. Transfection of miRNA-142-5p into HT-29 cells promoted cell apoptosis. Taken together, these results validated the feasibility of employing miRNAs in cancer treatment and highlighted the amazing diversity of miRNA-based therapeutic methods as a result of the wide range of oncogenic pathway effectors that can be targeted.

## 5. Outlook

miRNAs have continued to be at the forefront of CRC research. Future directions would involve the systematic investigation of differential miRNA expression profiles in healthy individuals and patients with different stages of CRC, which would lead to increased understanding of CRC pathogenesis. Successful application of miRNAs in CRC therapeutics also hinges on the development of viable delivery tools that can achieve targeted administration of miRNA activators or inhibitors to tumor tissues. In all aspects, miRNAs-based diagnostic and treatment strategies will play increasingly pivotal roles in improving the outcome of CRC patients in the near future.
